# Efficacy of an Online Physical Activity Intervention Coordinated With Routine Clinical Care: Protocol for a Pilot Randomized Controlled Trial

**DOI:** 10.2196/18891

**Published:** 2020-11-03

**Authors:** Bonny Rockette-Wagner, Gary S Fischer, Andrea M Kriska, Molly B Conroy, David Dunstan, Caroline Roumpz, Kathleen M McTigue

**Affiliations:** 1 Department of Epidemiology School of Public Health University of Pittsburgh Pittsburgh, PA United States; 2 Department of Medicine University of Pittsburgh Pittsburgh, PA United States; 3 Division of General Internal Medicine University of Utah Salt Lake City, UT United States; 4 Baker Heart and Diabetes Institute Melbourne Australia

**Keywords:** physical activity, online intervention, clinical translational research

## Abstract

**Background:**

Most adults are not achieving recommended levels of physical activity (150 minutes/week, moderate-to-vigorous intensity). Inadequate activity levels are associated with numerous poor health outcomes, and clinical recommendations endorse physical activity in the front-line treatment of obesity, diabetes, dyslipidemia, and hypertension. A framework for physical activity prescription and referral has been developed, but has not been widely implemented. This may be due, in part, to the lack of feasible and effective physical activity intervention programs designed to coordinate with clinical care delivery.

**Objective:**

This manuscript describes the protocol for a pilot randomized controlled trial (RCT) that tests the efficacy of a 13-week online intervention for increasing physical activity in adult primary care patients (aged 21-70 years) reporting inadequate activity levels. The feasibility of implementing specific components of a physical activity clinical referral program, including screening for low activity levels and reporting patient program success to referring physicians, will also be examined. Analyses will include participant perspectives on maintaining physical activity.

**Methods:**

This pilot study includes a 3-month wait-listed control RCT (1:1 ratio within age strata 21-54 and 55-70 years). After the RCT primary end point at 3 months, wait-listed participants are offered the full intervention and all participants are followed to 6 months after starting the intervention program. Primary RCT outcomes include differences across randomized groups in average step count, moderate-to-vigorous physical activity, and sedentary behavior (minutes/day) derived from accelerometers. Maintenance of physical activity changes will be examined for all participants at 6 months after the intervention start.

**Results:**

Recruitment took place between October 2018 and May 2019 (79 participants were randomized). Data collection was completed in February 2020. Primary data analyses are ongoing.

**Conclusions:**

The results of this study will inform the development of a clinical referral program for physical activity improvement that combines an online intervention with clinical screening for low activity levels, support for postintervention behavior maintenance, and feedback to the referring physician.

**Trial Registration:**

ClinicalTrials.gov NCT03695016; https://clinicaltrials.gov/ct2/show/NCT03695016.

**International Registered Report Identifier (IRRID):**

DERR1-10.2196/18891

## Introduction

More than 20 years ago, the Surgeon General’s report on physical activity summarized the health effects of inadequate physical activity, suggesting that inadequately low activity was associated with an increased risk for numerous poor health outcomes, including all-cause mortality, cancer, cardiovascular disease, and type 2 diabetes [1,2]. The report concluded with a recommendation for all adults to achieve at least 150 minutes of moderate-to-vigorous physical activity (like brisk walking) each week [3]. Unfortunately, less than half of US adults are currently meeting this recommendation [4-7], and the existing prevalence of inadequate activity levels has resulted in an estimated US $53 billion in health costs/year worldwide [8].

Clinical recommendations from the American Heart Association, the American Diabetes Association, and the US Preventive Services Task Force endorse physical activity in the treatment of common health problems, including obesity, diabetes, dyslipidemia, and hypertension [9-12]. It has been suggested that clinical referral for increasing physical activity has the potential to improve patient outcomes and lower health care costs [13-15]. Furthermore, advice from clinicians has been shown to greatly influence lifestyle behaviors and increase patient satisfaction with clinical care [16,17]. However, treating low activity levels is typically not currently part of routine clinical care [18-21]. Commonly cited reasons for this omission in routine care include lack of physician education and training on activity recommendations or referral options and lack of time and resources [22,23].

To enhance the incorporation of physical activity assessment and referral in standard disease prevention and clinical treatment, in 2007, the American College of Sports Medicine and the American Medical Association launched the Exercise is Medicine (EIM) initiative [13,24]. The EIM initiative provides support for clinical referral to physical activity by providing a framework and materials for physicians (eg, educational materials on physical activity recommendations, sample conversations with patients, and physical activity prescription pads) to facilitate a dialogue between providers and patients regarding physical activity participation.

**Figure 1:**
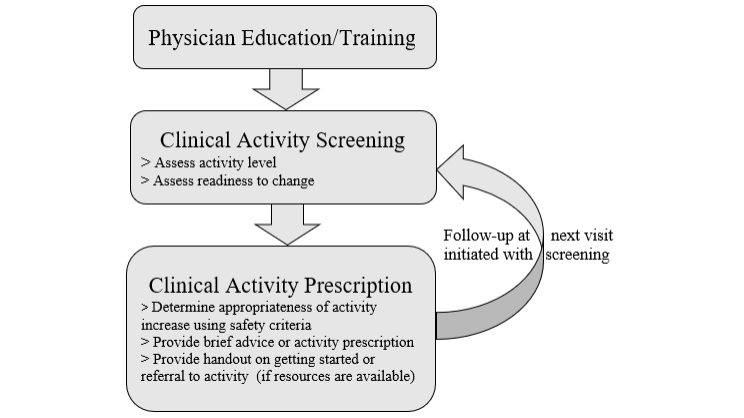
Existing process for physical activity prescription and referral, based on the American College of Sports Medicine’s Exercise is Medicine program.

EIM-based programs typically involve identification of patients with low activity levels using a simple questionnaire, advice from a health professional to increase physical activity, and suggestions for follow-up inquiries at the next routine visit (Figure 1) [13]. Clinical advice on physical activity can also be reinforced by referral to an exercise professional in the community or to an evidence-based physical activity intervention program [25]. However, published studies suggest that most existing programs involving clinical referral to physical activity for generally healthy adults are limited to physical activity screening and brief clinical advice to increase physical activity with a physical activity prescription, but without problem-solving support, gradual goal setting, or follow-up contact between visits [13,25-28], despite the fact that these are key components of effective behavioral change programs [13,25].

In addition to addressing factors that are relevant to the clinical setting, physical activity programs for patient populations should incorporate evidence-based aspects of successful behavior change programs [13,14,24]. Yet, recent meta-analysis findings suggested that only three of the 13 referenced clinical physical activity programs included theory-driven behavioral interventions and only one of the three had more than three sessions [25,29-31]. Furthermore, no programs provided support for maintenance of physical activity behavior change. This is despite the fact that the Centers for Medicare & Medicaid Services (CMS) is now reimbursing lifestyle interventions based on that developed and evaluated as part of the Diabetes Prevention Program (DPP). These highly successful translations of the theory-driven DPP behavioral intervention include weight loss and physical activity goals. These programs typically offer 12 to 16 core sessions that include key components of social-cognitive theory followed by a maintenance phase [32-34]. The lack of both theory-driven behavior change strategies and support following an initial behavior change intervention may explain the lower success with participants maintaining physical activity that has been reported for clinical physical activity programs, compared with programs delivered in other settings (community, workplace, and university) [25].

This manuscript describes the protocol for the ActiveGOALS Study. The primary aim of the study is to implement and evaluate an EIM-based approach that builds on successful translational lifestyle interventions [14,35,36]. This study involves developing and piloting the behavior change core intervention sessions (first 3 months) of a proposed 1-year intervention program. In contrast to traditional EIM programs, this program will include an internet-based physical activity intervention for the general adult patient population with remote coach support that is rooted in social-cognitive theory.

## Methods

### Study Design Overview

The ActiveGOALS Study was designed to develop, implement, and evaluate a 3-month, one-on-one, online intervention for promoting behavior change related to physical activity improvement in adult primary care patients (study sample of 80 participants, including 40 aged 21-54 years and 40 aged 55-70 years) with low activity levels using a randomized controlled trial (RCT) design with a wait-listed control group. Patients with low activity levels were recruited through several means, including self-referral and referral by their physician. Participants were required to be able to perform unsupervised moderate-intensity physical activity (eg, brisk walking), with a physician referral form required to confirm eligibility.

**Figure 2:**
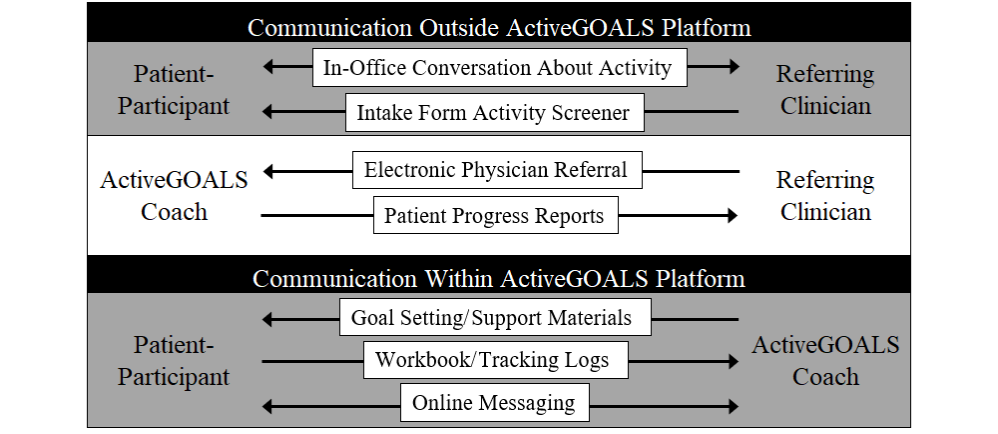
ActiveGOALS program delivery process showing communication between the providers and patient (arrows indicate the direction of communication for each activity).

**Figure 3:**
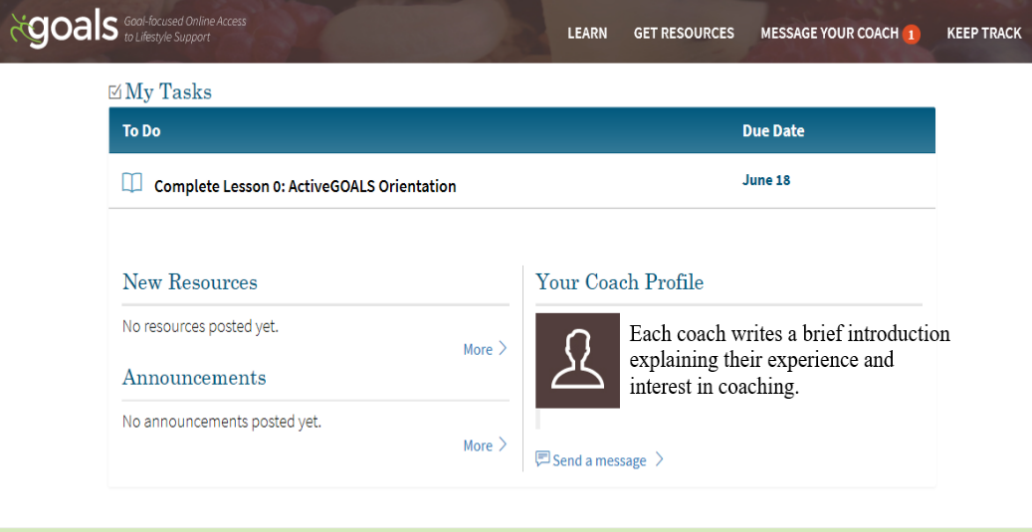
Screenshot of the ActiveGOALS intervention platform home page.

The use of an online platform improves convenience for patients and facilitates communication among the patient, referring physician, and ActiveGOALS coach (Figure 2). Patient participants accessed all ActiveGOALS program materials (13 weekly sessions, tracking tools, and workbook pages) through the online platform (Figure 3). Trained coaches tracked participant progress and communicated with participants through a secure messaging system within the platform to provide brief weekly advice and support or to answer questions related to physical activity. Participant progress was also reported to referring physicians.

**Figure 4:**
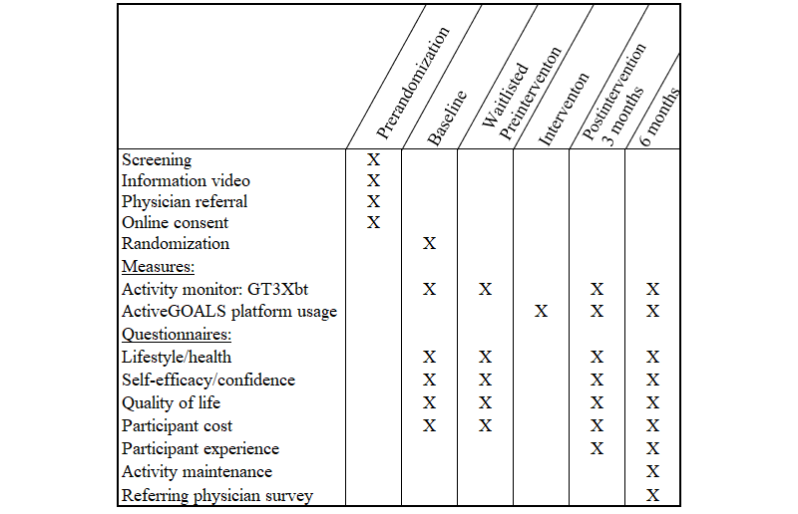
ActiveGOALS study timeline (only the wait-listed control group had the second preintervention assessment).

All enrolled participants were randomized to receive the intervention immediately (*immediate*) or after a 3-month waiting period (*wait-listed*). Assessments for the RCT were conducted at baseline and after 3 months when the *immediate* participants should have completed the 13 weekly ActiveGOALS program sessions and before the *wait-listed* participants were offered the ActiveGOALS program. We hypothesized that participants randomized to receive intervention immediately would have much larger (1) increases in steps/day and moderate-to-vigorous physical activity (minutes/day) and (2) decreases in sedentary behavior (minutes/day) between baseline and 3 months when compared to the wait-listed control group participants. To evaluate whether the effects of the intervention lasted beyond the weekly sessions, all participants were followed for 6 months after the start of their intervention (Figure 4). This study was approved by the Institutional Review Board at the University of Pittsburgh (STUDY19080212).

### Patient-Centered Outcomes Research

We used a patient-centered research approach, engaging key stakeholders as members of the research team, using PaTH Clinical Research Network stakeholder engagement resources [37,38]. The research team included a patient partner and a community-based certified exercise specialist with an interest in helping others develop healthier lifestyles. The study partners were invited to participate in research meetings, and they provided guidance on intervention design and delivery, as well as the need for any additional patient-centered outcomes. They were compensated for each meeting attended.

Physician referral that included a confirmation that moderate physical activity was medically appropriate for the patient was required for study participation. A new physician referral form was required before returning to the program if an injury, illness, or surgery was reported during the study. Referring physicians also received two patient progress reports. The first progress report informed physicians of the participant’s reason for joining the program, participant’s baseline activity levels, program engagement (such as logging in, session completion, and tracking activity), and participant’s success in meeting the goals in the first half of the program. The second progress report provided information on engagement throughout the program and included graphs of average step counts and time spent in moderate-to-vigorous activity for the participant in each week of the program. Referring physicians were also mailed a brief five-question survey regarding the utility of the physician reports.

### Study Procedures

#### Intervention Development

ActiveGOALS intervention materials were adapted from existing lifestyle intervention materials involving social-cognitive behavior change theory and related strategies specific to supporting physical activity change and reducing sedentary behaviors, including barrier recognition and problem solving [14,36,39]. Materials were further refined with input from key stakeholders and assembled into structured lessons with interactive workbook pages (20-25 minutes long). Two technical lessons were also included, which provided help with using the ActiveGOALS platform and tracking, inputting, and viewing activity data in ActiveGOALS.

In all, 11 of the 13 intervention sessions were modified from the existing GOALS (Guided Online Access to Lifestyle Support) lifestyle intervention materials. The GOALS materials were developed as an individually focused online translation of the DPP, and like the DPP, it provided support for weight loss and physical activity goal achievement (150 minutes of at least moderate intensity physical activity per week) [14,35,40]. The GOALS platform was designed to be modified for use with lifestyle interventions that may have healthy lifestyle program goals not included in the original GOALS program.

For this study, GOALS sessions were redesigned to focus specifically on increasing physical activity to 150 minutes/week of moderate-to-vigorous activity, increasing daily step counts, and reducing daily sedentary time (without a weight-loss goal). In order to achieve the goal of reducing sedentary time, participants were guided to take short breaks from sedentary behaviors throughout the day as well as to take larger (10 minutes or more) breaks from sedentary behavior several times a day. Social-cognitive theory–based strategies, including barrier recognition and problem solving, were retained. A session on reducing sedentary behavior and tracking tools for sedentary time were adapted from materials developed for the Group Lifestyle Balance Program, an in-person group-based intervention developed for community use (CDC recognized/CMS reimbursable) by members of the team that created the original DPP lifestyle intervention [36,39,41]. Finally, one session on *maintaining physical activity while traveling* was developed specifically for ActiveGOALS. The modified intervention materials were presented through the online platform and delivered along with self-monitoring tools, links to reputable web-based materials that may support physical activity, and personalized e-coaching.

#### Participant Eligibility and Recruitment

To reduce participant burden, the study was designed to be conducted remotely, with no in-person participant visits. Study information was conveyed to potential participants by phone, through email, and via an online video describing the study. Recruitment took place between October 2018 and May 2019 through a local primary care office (University of Pittsburgh Physicians-General Internal Medicine-Oakland [UPP-GIMO], Pittsburgh, Pennsylvania) and through an online recruitment tool “Pitt+Me.” The Pitt+Me system is a registry of over 200,000 patient volunteers interested in participating in research studies. A continuous (rolling) enrollment strategy was used to recruit men and women aged 21 to 70 years.

Women currently pregnant or planning a pregnancy in less than 6 months and individuals who were nonambulatory or planning a procedure that would cause them to be nonambulatory in less than 6 months were not eligible. A sixth grade literacy level and access to a computer and the internet were required for participation. Ability to safely perform physical activity at a moderate intensity (like a brisk walk) for bouts of 10 minutes without direct supervision was required (per both participant self-report and primary care physician referral).

We aimed to recruit a sample that reflects the general patient population within UPP-GIMO for sex (68% female) and race/ethnicity (25% minority; mostly African American people). No individual was excluded from the study on the basis of race or gender. Some previous studies suggested that readiness to change and technology adoption may differ between older and younger adults [42,43]. Although there were no targeted recruitment strategies by age group, to guarantee participation by older and younger adults, recruitment was stratified by age group (80 participants, including 40 aged 21-54 years and 40 aged 55-70 years).

Recruitment strategies at UPP-GIMO included the implementation of a two-question physical inactivity screener [44], which was used to identify potential participants (those reporting <150 minutes of planned physical activity per week) at the beginning of annual physical visits. The ActiveGOALS Study principle investigator also attended a UPP-GIMO departmental physician meeting and introduced the study to attending physicians and clinical staff. Study fliers and brochures were distributed in the waiting room and exam rooms at UPP-GIMO. Patients with low activity levels could ask their physicians for a study referral at their office visit or could reach out to the study directly to complete the screening before reaching out to their physicians. Physicians were also encouraged to identify potentially eligible patients, confirm patient interest, and refer them to the study. Referrals were mailed or faxed to study staff (within UPMC, referral could take place via a standard electronic referral through the Epic Electronic Health Record [EHR]).

Study materials were posted to the Pitt+ME online portal and a targeted email was sent out to approximately 3500 adults aged 21 to 70 years, who expressed interest in lifestyle programs. This targeted email provided study information and a link to the portal where interested individuals could be prescreened by Pitt+ME staff. Interested or eligible individuals were referred to ActiveGOALS Study staff for confirmatory phone screening. Individuals without a primary care physician to complete the required referral, could be referred to UPP-GIMO (if they reported having insurance), student health (if they reporting being a student at a local university or college), and/or a local free clinic (if they reported having no insurance).

#### Screening

After referral by EHR or Pitt+ME, screening for eligibility was conducted by phone, during which participants were asked to verify their activity levels and answer a series of questions to determine eligibility. These questions included a three-question disability screener that has been previously used in conjunction with activity assessments to identify individuals with disability that could affect their ability to safely ambulate [45]. Additional safety screening was not required owing to the acquisition of a physician referral prior to enrollment.

Individuals were informed whether they were eligible for the program, provided with detailed study information at the end of the screening call, and given time to ask questions. Eligible and interested individuals were emailed a link to a short informational video, a copy of the consent form to read over, and a doctor’s referral form that they were required to have signed. The brief video provided enrollment requirements, the study purpose/history, and information on program expectations.

#### Consent and Randomization

Study staff followed-up with the interested/eligible participants prior to consent to answer questions. Only individuals who returned a doctor’s referral were forwarded a personalized link to an online consent form developed using REDCap, a Health Insurance Portability and Accountability Act (HIPAA) compliant web application for building and managing online surveys and databases. Participants were randomized 1:1 in REDCap to receive the ActiveGOALS intervention immediately or after a 3-month waiting period. Block randomization in groups of four was conducted within each age strata. Study staff did not have access to the assignment list. Staff were required to verify completeness of a record before the randomized group assignment would become visible in REDCap. Each participant was informed of their assignment as soon as it became known to the staff.

#### Intervention Delivery

The ActiveGOALS intervention was accessed online. The weekly behavior change curriculum was presented through the online platform. Participants were also provided with self-monitoring tools and links to reputable web-based physical activity materials. A trained lifestyle coach with a background in exercise physiology or physical activity and health communicated weekly with participants via a secure messaging system. They also provided feedback on participant workbook pages and worked with participants to set goals and develop individualized strategies for increasing and maintaining physical activity levels.

Participants were given a body-worn step counter as an intervention tool. Participants randomized to immediate intervention were given an Omron Alvita monitor. To examine whether a monitor with additional features might add to the success of the program (examined as change in activity and program satisfaction), after the RCT ended and the wait-listed participants were offered the full ActiveGOALS program, they were given a Fitbit Alta monitor instead of the Omron monitor. They could also print tracking logs for recording time spent in moderate-to-vigorous activities and breaks from sedentary behavior. All tracked activity was entered by hand into the online intervention platform.

Additional contact was used to promote adherence to the intervention and ensure high rates of follow-up. Participants who did not log in for over 14 days received an extra message in ActiveGOALS from their coach. If there was no response within several days, the coach would send an email or call the participant. Calls were used to re-establish contact and were not used as a supplementary coaching tool. Postcards could be sent via postal mail if, after 21 days, there was still no contact with the participant. Postcards were also sent to acknowledge milestone achievement. After sessions were completed, participants retained access to the ActiveGOALS platform, including completed session materials, tracking software, and supplementary materials (until after their final study assessment). However, coaching support was no longer provided.

#### Wait-Listed Control Group

Participants randomized to the wait-listed control group received the full ActiveGOALS intervention program after a 3-month waiting period. During the waiting period, they received monthly health fliers on general health topics unrelated to the ActiveGOALS intervention (sleep and hydration). The *wait-listed* participants also had one additional preintervention assessment (physical activity monitoring and online questionnaires).

#### Outcome Measures

To ensure accurate and objective measurement of physical activity, the outcomes of moderate-to-vigorous physical activity (minutes/day), steps/day, and sedentary minutes/day were assessed with ActiGraph GT3Xbt research grade monitors. The primary outcome of interest was the change between baseline and the 3-month postbaseline follow-up visit (postintervention for the *immediate* group and the second preintervention for the *wait-listed* group; Figure 4).

Participants were mailed a monitor and received video and paper instructions for wearing an ActiGraph accelerometer on their waist at each assessment time point. They were instructed to wear the monitor during waking hours for the 10 days following receipt of the monitor. Based on previous research, a minimum of 4 days of recording with at least 10 hours of wear time is required for an accelerometer recording to be representative of a person’s “typical” activity levels [46].

For the examination of secondary outcomes related to program success over 6 months, additional assessments were conducted for all participants at 3 and 6 months after program start (Figure 4). At all assessments (pre- and postintervention participation), participants were emailed a link providing access to an online portal to complete the lifestyle/medical/weight, self-efficacy/confidence, quality of life (European Quality of Life Visual Analogue Scale [EQVAS] from the European Quality of Life 5 Dimension [EuroQol5D] questionnaire and the Promis-29 questionnaire) [47,48], and program cost survey questionnaires. All questionnaires have been previously validated [36,47-49].

A participant satisfaction survey was also provided at the 3-month postprogram assessment (within 2 weeks of completing the 13-week program). The questionnaire was designed to provide feedback on patient-centered aspects of program utility/success and focused on the ActiveGOALS platform, coaching, session materials, and tracking/goal-setting materials. Questions developed to identify facilitators and barriers to program usage and success were also included.

Questions related to the maintenance of physical activity were given to participants with their final assessment (approximately 3 months after the 13-week program was completed) toward informing the design of physical activity maintenance session materials that would follow the existing 13 sessions. Participant perceptions of activity maintenance were assessed, along with their general attitudes and opinions toward their ability to maintain their current activity levels.

To minimize missing data, participants were alerted to incomplete answers in REDCap. Project staff members checked all questionnaires for completeness, notified participants via email if a questionnaire was not complete, and provided a link to complete the questionnaire.

Research staff communicating with participants regarding assessments were blinded to patient assignment. To ensure high rates of follow-up, participants received email reminders of each outcome assessment plus mail or telephone reminders if needed (up to five contacts). Reasons for missing data and participant withdrawal or drop-out were collected. Additional outcomes will include information from the ActiveGOALS platform related to lesson completion and tracking physical activity and sedentary breaks.

#### Incentives

Participants were compensated US $20 for completing each assessment (wearing a research activity monitor for 10 days and completing online questionnaires). Compensation was not provided for participation in the ActiveGOALS program (eg, session completion, tracking of activity, contacting their coach, and meeting physical activity goals).

#### Power and Sample Size

This pilot study was powered with enrollment of 80 participants (assuming approximately 20% attrition; n=64) to identify mean differences between randomized groups at 3 months (two-sided; *P*<.05) of 5 minutes/day moderate-to-vigorous physical activity (β=.97), 1500 steps/day (β=.92), and 75 minutes/day sedentary behavior (β=.82). Reference mean (SD) values were calculated from available baseline waist worn accelerometer data for participants with below recommended activity levels in another study being conducted by members of this study group.

### Analyses

To ensure rigor and reproducibility, the primary analyses will be based on between-group comparisons for the 3-month RCT data (intention-to-treat), and linear mixed effects regression models will be used to assess differences across treatment groups (immediate intervention and wait-listed control) for primary outcomes collected from the accelerometers. Secondary analyses to inform the development of additional “maintenance phase” program materials (pre- to postintervention changes across 0, 3, and 6 months) will be conducted. Linear mixed models will be used to determine relationships between sessions completed, time to program completion, tracking frequency from the ActiveGOALS platform, quality of life, self-efficacy and confidence, and self-reported weight and the primary study outcomes. These results will be analyzed and presented for all participants combined, controlling for the study arm.

Comparisons across important subgroups, including randomized assignment, and across the two age groups (21-54 and 55-70 years) will be conducted to assess differences in change across groups for all outcomes. Results may also be examined across other important subgroups identified during analyses (although subgroup analyses may be fully powered). Descriptive statistics on patient satisfaction and cost will be determined. An evaluation of missing data will be conducted to determine the amount/type of missing data. Based on the findings, sensitivity analyses will be conducted using an appropriate imputation method (mean of other group or multiple imputation).

## Results

Data collection for this study has been completed. Processing data from the activity monitors is underway, after which analyses will begin. The main study results will be submitted for publication in 2021. No adverse events were reported.

**Figure 5:**
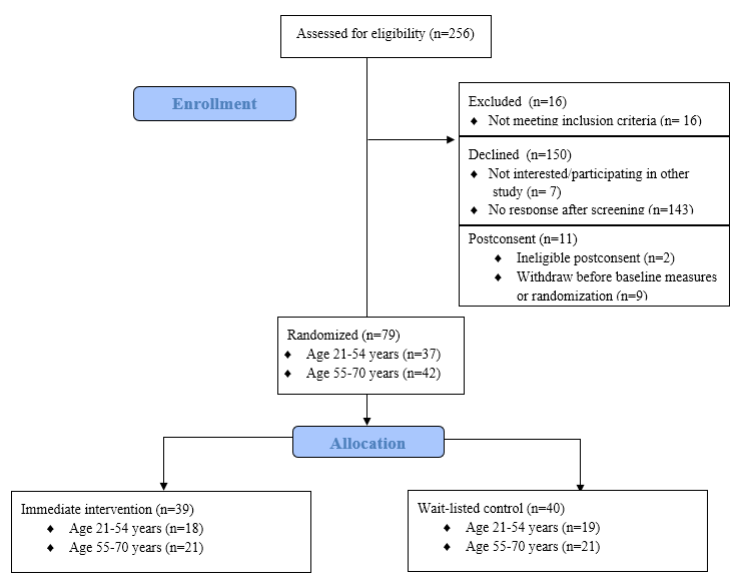
Recruitment flow chart.

A total of 256 individuals were screened for eligibility (Figure 5). Few were determined ineligible during phone screening (n=16). Reasons for ineligibility included self-report of meeting recommended activity levels, no desire to involve a physician, no dependable access to a computer, and inability to safely perform moderate-intensity activity. Seven individuals declined to participate owing to one of the following reasons: considering participation in another study with physical activity goals and no interest in the program format. An additional 143 individuals did not contact study staff during the 5 months of active recruitment and could not be reached after three attempts.

Physician referrals and signed consent forms were received for 90 individuals (Figure 4). Two individuals signed the consent while participating in other lifestyle programs with problem solving and goal setting for physical activity, making them ineligible. Another nine individuals decided not to participate in the study shortly after consenting but before completing the baseline assessments or being randomized. Reasons for withdrawing after consent included new diagnosis requiring surgery, need to care for a family member, and no longer wanting to participate. A total of 79 individuals (37 aged 21-54 years and 42 aged 55-70 years) completed baseline assessments and were randomized for the study.

The mean age of the participants at baseline was 50.8 (SD 15.8) years. Overall, 77% (61/79) were female and 24% (19/79) reported a race/ethnicity other than white, non-Hispanic (approximately 15% African American, non-Hispanic). Our recruitment goal was to recruit a sample representative of the UPP-GIMO patient population, which is 68% female and 25% other than white, non-Hispanic.

Based on self-reported activity levels from the study screener, median physical activity levels were 15 (IQR 0-43) minutes/week of at least moderate-intensity physical activity. A total of 56 of 79 (71%) participants reported overweight or obese BMI (≥25 kg/m^2^) at baseline. Additionally, most participants (58/79, 73%) reported attending “some college” or completing a degree, and 39% (n=31) reported working full time (≥40 hours/week), 17% (n=13) reported preretirement part-time work (<40 hours/week), 11% (n= 9) reported unemployment (preretirement), and 33% (n=26) reported partial or full retirement.

**Figure 6:**
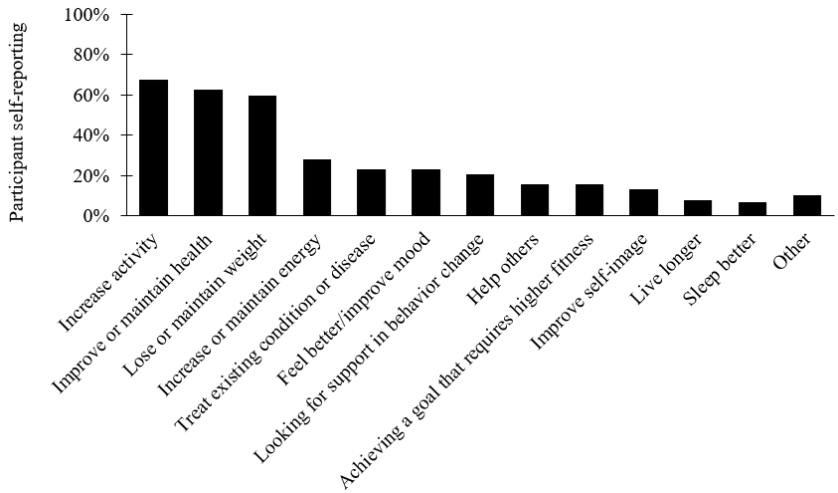
Self-reported reasons for taking part in the ActiveGOALS Study intervention (percentages based on 79 participants).

At the beginning of the program, all participants were prompted to “list your reasons for joining the program.” Participants were able to provide up to six reasons for wanting to take part in the ActiveGOALS Study intervention. The enrollment reasons were coded by two readers and summarized by percentage of participants reporting at least one reason pertaining to that category (Figure 6).

In general, participants wanted to be more physically active and hoped to see improvements in health toward preventing diseases, increasing longevity, and/or treating existing conditions. Specifically, participants most commonly reported wanting to increase activity (n=53, 67%), wanting to improve or maintain health (n=49, 62%), and wanting to lose or maintain weight (n=47, 60%). Not all reasons for participation were directly related to health. Close to 15% (n=12) of participants reported having specific life goals that required improving their fitness. Similarly, close to 15% (n=12) wanted to help others through their participation (most commonly reporting that they wanted to set a good example for others).

## Discussion

### Brief Summary

The ActiveGOALS Study was designed to determine the efficacy of an online physical activity intervention designed to coordinate with a patient’s clinical care. The study was also designed to examine maintenance of physical activity changes for up to 3 months after program session completion and to determine patient attitudes and beliefs regarding maintenance of behavior changes related to physical activity. The results of this study will be utilized for the development of a referral program for physicians to refer primary care patients with low activity levels to a year-long intervention program for physical activity improvement.

### Limitations

Currently, there are no guidelines suggesting a clinically relevant maximum level of sedentary behavior that should be observed by adults or a specific number of minutes to set as a goal for reducing sedentary behavior [10,50]. This study was powered for a sedentary reduction of 75 minutes/day, which was suggested to be feasible in a recent meta-analysis (although most studies set lower goals and achieved 30 to 60-minute reductions) [51]. Therefore, it is possible that this study could be underpowered for a smaller level of change that is later identified as a clinically relevant sedentary reduction goal.

The generalizability of this study to other populations may be limited by the fact that the study sample was recruited in Pittsburgh and the surrounding region. For example, our study sample predominantly involved white, non-Hispanic people, with African American, non-Hispanic people reported as the next largest racial/ethnic group. There were few individuals reporting other racial or ethnic groups.

### Strengths

This study has a number of important strengths. First, health centers in Pittsburgh, Pennsylvania draw patients from a wide radius, and the online research format allowed us to recruit participants from within and beyond the Pittsburgh metro area. It also made it possible to retain participants who moved away from Pittsburgh and/or changed health providers during the study period.

The outcome measures for this study were collected with a validated waist-worn accelerometer. Most existing studies examining the effects of clinical prescription programs for physical activity improvement and lifestyle interventions with physical activity goals rely on self-report questionnaire data [13,25,32]. While questionnaires are useful for providing information on the types of activities performed, they can be subject to misreporting bias and are not as valid as accelerometers for providing precise estimates of *total time* spent performing physical activity or in sedentary behavior [52-54].

Additionally, existing studies for clinical physical activity referral are typically limited to clinical advice [25]. For the ActiveGOALS intervention program, we combined evidence-based strategies for behavioral change with input from important stakeholders toward developing materials that provide patients with support for behavior change in a way that is both feasible and acceptable for adult clinical patient populations. We contend that patient- and provider-centered approaches are needed to identify and address needs specific to clinical physical activity referral, which may not be as important to programs delivered in other settings. This study is also unique in reporting patient progress to referring physicians and collecting feedback from physicians regarding the utility of patient reports.

Finally, clinical physical activity referral programs currently lack strategies for long-term maintenance of behavior change. By collecting data on maintenance of behavior changes and participant attitudes and beliefs regarding long-term maintenance of physical activity, we will be able to develop better strategies for postintervention physical activity maintenance phase materials.

### Conclusions

ActiveGOALS program materials were developed using existing evidence-based materials and inputs from important stakeholders. To our knowledge, there are few theory-based programs involving clinical referral to physical activity and none involving a full social-cognitive theory–based curriculum with problem solving and gradual goal setting [25,29-31]. Furthermore, owing to short follow-up periods and the lack of maintenance strategies in existing clinical programs for physical activity referral, little is known about behavior maintenance following participation in clinical referral programs [25].

The results will be used to inform the development of a 12-month theory-based behavioral change program for physical activity improvement that will be coordinated with a patient’s clinical care. This program will include both behavior change and maintenance strategies, as well as clinically administered screening for low activity levels, electronic referral to the intervention, and meaningful feedback on participant progress to referring clinical teams.

## Appendix

Multimedia Appendix 1 K12 reviewer comments and author addendum with reported changes made per reviewer comments.

## Abbreviations

DPP: Diabetes Prevention Program
EHR: electronic health record
EIM: Exercise is Medicine
GOALS: Guided Online Access to Lifestyle Support
RCT: randomized controlled trial
UPP-GIMO: University of Pittsburgh Physicians-General Internal Medicine Oakland
