# Supplementary material for: Efficacy of an Online Physical Activity Intervention Coordinated With Routine Clinical Care: Protocol for a Pilot Randomized Controlled Trial
Source: JMIR Res Protoc. 2020 Nov 3;9(11):e18891. doi: 10.2196/18891 (PMC7671848; doi:10.2196/18891)
Supplement: Multimedia Appendix 1 [file resprot_v9i11e18891_app1.pdf]

The following is a compilation of the comments made by the reviewers in respect to your application to the Clinical and Translational Science (CTS) Scholars Program (K12) at the Institute for Clinical Research Education (ICRE) at the University of Pittsburgh. No decisions have been made yet; however, we wanted you to have the comments.

Reviewer #1

OVERALL IMPACT:

Strong, productive candidate. HAs developed an expertise in PA assessment. Looking to expand her understanding of clinical translational research by 1) gaining experience working with technology-enabled research and intervention tools, 2) designing and implementing PA interventions, and 3) building basic skills in qualitative and mixed methods. Comprehensive training plan that ties nicely with career development goals and research plan. Excellent mentors and strong environment. Interesting research project that may be overly ambitious. I believe project could be difficult to pull off. Would like to see candidate address potential pitfalls and barriers and how she would address them.

RESEARCH PLAN:

<u>Strengths:</u>

well defined, important and significant project. Laid out in a systematic way. Defines deliverables of the project. There is a need/interest/relationship with partners that are important for the project.

<u>Weaknesses:</u>

ambitious
presented as this will be a very easy thing to do..... I would have liked to see the candidate address what the potential barriers and pitfalls and what might be some potential solutions. I think the project is going to be hard to pull off.

ADDITIONAL COMMENTS:

<u>Strengths:</u>

<u>Weaknesses:</u>

Reviewer #2

OVERALL IMPACT:

Overall, this is a strong candidate who is well trained and has excellent mentors. However, her research plan has significant weaknesses, which limits the enthusiasm for this proposal.

RESEARCH PLAN:

<u>Strengths:</u>

Providing online PA intervention is novel in a primary care practice among sedentary patients. Feeding this information back to the provider through the EHR is innovative as it can be used to encourage the patient to continue with the PA. The study proposes recruiting participants from a primary care practice through a variety of methods (directly from the clinic, Pitt+Me, etc).

<u>Weaknesses:</u>

- The age range is quite large (21-70). with such a large span and a limited sample size, it will be difficult to study the results by age category. It seems reasonable that what would work for a 21 year old would be categorically different than a 70 year old.
- The intervention includes the use of Fitbit, which will be difficult to know what had the most impact - the intervention or the Fitbit (which provides direct feedback on activity.)
- "Participant cost survey" is included, but it is unclear what this is. Are there participant costs?
- It is unclear why a Fitbit is needed and ActiGraph is needed.

ADDITIONAL COMMENTS:

<u>Strengths:</u>

<u>Weaknesses:</u>

Reviewer #3

OVERALL IMPACT:

The applicant's track record demonstrated potential to become an independent and productive researcher with a unique skill set. The proposed intervention is significant and innovative, but the trial will benefit from having a trial statistician. There are some minor issues in approach, but they are all addressable.

RESEARCH PLAN:

<u>Strengths:</u>

1. The proposed intervention is unique in the sense that its focus is on reducing sedentary behavior rather than increasing intense activity.
2. Idea of integrating activity monitor data to provide feedback to the provider is significant and innovative.

<u>Weaknesses:</u>

1. There is no description about randomization process. It seems stratification by age group might be needed because of the small sample size and the technological aspect of the intervention. Older adults may not benefit from the intervention as much as tech-savvy young adults.
2. Sample size calculation did not specify assumed variation of the data, making it impossible to replicate the calculation to verify.
3. In statistical analysis plan, comparison of baseline data is missing. Although the randomization is supposed to make the two groups balanced, they can be unbalanced by chance especially when the sample size is small. Thus comparison of the baseline data is necessary to make sure the groups are comparable.

ADDITIONAL COMMENTS:

<u>Strengths:</u>

<u>Weaknesses:</u>

From Authors: This grant was funded. Most of the reviewers' comments referred to issues of clarity due to wording limitations in the proposal. However, the following changes were made to the research design as a result of the reviewers' comments.

1.The program requires both a validated, research outcome measure of physical activity and an intervention measure of activity that provides continuously available output to participants. Unfortunately, the most validated research monitors are not as feasible for long term daily wear by participants and do not provide output to the participant- to reduce reactivity. Therefore, the program requires the use of a second monitor that was designed for individuals to use as a personal step count tracker. To remove any concern of interference by goal setting materials provided by a Fitbit monitor, we gave the immediate intervention participants a more basic tracker, the Omron Alvita monitor. The Omron monitor is a simple waist worn monitor for tracking steps that does not have push messaging or any goal setting software. Therefore, working only to track steps as part of the designed ActiveGOALS intervention. However, so that we could examine the question of whether the use of a monitor with additional features might add to the success of the program, after the randomized control trial portion of the study had ended and the wait-listed participants were offered the full ActiveGOALS program they were given a Fitbit Alta monitor instead of the Omron monitor. Questions were added to the 3 and 6 month follow-up questionnaires regarding the utility of the activity monitor that was provided with the program. These results, along with difference in changes in activity, will be compared between the two groups to provide pilot data on whether a more advanced or simplistic step counter appeared to be more helpful to participants.

2. Due to concerns over differences in program uptake, for an online program by adults of different ages, we stratified recruitment by age group 21-54 and 55-70. This ensured that we would be able to examine uptake in both age groups and that our two randomized groups would have equal numbers of older and younger adults.
